# *FZP* determines grain size and sterile lemma fate in rice

**DOI:** 10.1093/jxb/ery264

**Published:** 2018-07-19

**Authors:** Deyong Ren, Jiang Hu, Qiankun Xu, Yuanjiang Cui, Yu Zhang, Tingting Zhou, Yuchun Rao, Dawei Xue, Dali Zeng, Guangheng Zhang, Zhenyu Gao, Li Zhu, Lan Shen, Guang Chen, Longbiao Guo, Qian Qian

**Affiliations:** 1 State Key Lab of Rice Biology, China National Rice Research Institute, Hangzhou, P. R. China; 2 College of Chemistry and Life Sciences, Zhejiang Normal University, Jinhua, P. R. China; 3 College of Life and Environmental Sciences, Hangzhou Normal University, Hangzhou, P. R. China

**Keywords:** CRISPR-Cas9, dehydration-responsive element (DRE), *FZP*, GCC-box, grain size, rice (*Oryza sativa*)

## Abstract

In grass, the spikelet is a unique inflorescence structure that directly determines grain yield. Despite a great deal of research, the molecular mechanisms behind spikelet development are not fully understood. In the study, *FZP* encodes an ERF domain protein, and functions in grain size and sterile lemma identity. Mutation of *FZP* causes smaller grains and degenerated sterile lemmas. The small *fzp-12* grains were caused by a reduction in cell number and size in the hulls. Interestingly, the sterile lemma underwent a homeotic transformation into a rudimentary glume in the *fzp-12* and *fzp-13* mutants, whereas the sterile lemma underwent a homeotic transformation into a lemma in *FZP* over-expressing plants, suggesting that *FZP* specifically determines the sterile lemma identity. We confirmed the function of *FZP* by complementation, CRISPR-Cas9 gene editing, and cytological and molecular tests. Additionally, *FZP* interacts specifically with the GCC-box and DRE motifs, and may be involved in regulation of the downstream genes. Our results revealed that *FZP* plays a vital role in the regulation of grain size, and first provides clear evidence in support of the hypothesis that the lemma, rudimentary glume, and sterile lemma are homologous organs.

## Teaser

This study revealed that *FZP* functions in grain size and sterile lemma identity, and supported the hypothesis that the lemma, rudimentary glume, and sterile lemma are homologous organs.

## Introduction

Rice (*Oryza sativa* L.) is the important and staple food. Increasing rice grain yield provides the food demands and security of a rapidly growing population. Grain size and shape in rice (*Oryza sativa*) is specified by grain length, width, and thickness, which determine overall yield and quality. Multiple genetic factors control grain weight (Hu *et al.*, 2015, Huang *et al.*, 2017), but the underlying regulatory mechanisms that determine grain size and shape are still largely unknown.

The spikelet hull restricts grain growth and determines the final grain size in rice (Li and Li, 2016). Most of the identified genes and quantitative trait loci (QTLs) that influence grain size act through regulation of the proliferation and expansion of the cells in the spikelet hull. For example, *GRAIN SIZE 3* (*GS3*), *GRAIN LENGTH 3* (*qGL3/GL3.1*), and *BIG GRAIN 1* (*BG1*) determine grain size by controlling cell number. *GS3* encodes a putative G protein γ subunit, and its loss of function induces long grains as a result of increased cell number (Mao *et al.*, 2010, Fan *et al.*, 2006). *qGL3/GL3.1* encodes a phosphatase kelch protein and dephosphorylates its substrate cyclin-T1;3. Loss of function of *qGL3/GL3.1* produces longer grains by increasing cell number (Zhang *et al.*, 2012; Qi *et al.*, 2012). *BG1* encodes a novel membrane-localized protein and positively regulates auxin response and transport. Over-expression of *BG1* results in increased grain size and its knockdown leads to smaller grains by regulating cell number (Liu *et al.*, 2015). Several other factors that control grain size by influencing cell size have also been cloned, including *GRAIN LENGTH 2/GRAIN SIZE 2* (*GL2*/*GS2*), *Grain Length on Chromosome 7* (*GL7*), *POSITIVE REGULATOR OF GRAINLENGTH 1* (*PGL1*) and *PGL2*. *GL2*/*GS2* encodes the transcription factor GROWTHREGULATING FACTOR 4 (OsGRF4) and is regulated by the microRNA OsmiR396. Mutation in *GL2*/*GS2* disturbs OsmiR396-directed regulation of *GL2*/*GS2*, resulting in large grains predominantly by influencing cell size (Che *et al.*, 2016; Hu *et al.*, 2015). GL7 is homologous to *Arabidopsis thaliana* LONGIFOLIA, and upregulation of *GL7* expression produces slender grains as a result of longitudinally-increased cell length and transversely-decreased cell width (Wang *et al.*, 2015a, 2015b). Atypical helix-loop-helix proteins encoded by *PGL1* and *PGL2* affect grain length by regulating cell length (Heang and Sassa, 2012a, 2012b). The above studies revealed that cell proliferation and cell expansion play key roles in coordinately controlling grain size in rice.

In rice, the spikelet comprises one terminal fertile floret and unique glumes: sterile lemmas and rudimentary glumes, which is different from those of other grasses (Ren *et al.*, 2018). Despite a great deal of research, the origins of glumes are still debated. Recently, several genes have been cloned, which helped to elucidate the origins of these organs in rice. *LONG STERILE LEMMA* (*G1*) encodes a plant-specific gene family protein with an unknown domain. Mutation of *G1* causes the sterile lemma to develop lemma-like characteristics (Yoshida *et al.*, 2009). *OsMADS34* is an important regulator of the identity of sterile lemma (Kobayashi *et al.*, 2010; Gao *et al.*, 2010). The *osmads34* mutant shows enlarged glumes (sterile lemmas and rudimentary glumes) with lemma-like cellular structure. Similarly, the *dg1* mutant also displays elongated rudimentary glumes and sterile lemmas (Yu *et al.*, 2017). Furthermore, *DROOPING LEAF* (*DL*) signal was found in these abnormal glumes, indicating that both the sterile lemmas and rudimentary glumes had the lemma identity (Lin *et al.*, 2014; Ren *et al.*, 2016; Yu *et al.*, 2017). *ABERRANT SPIKELET AND PANICLE 1* (*ASP1*) and *EXTRA GLUME 1* (*EG1*) determine the identity of sterile lemma (Li *et al.*, 2009; Yoshida *et al.*, 2012). In the *asp1* and *eg1* mutants, the elongated sterile lemma is caused by homeotic transformation of the sterile lemma into the lemma, and the *asp1* mutant also displayed elongated rudimentary glumes, resembling the sterile lemma in size and structure. *OsMADS1* plays a role in the identity of the lemma and palea, and its ectopic expression in sterile lemmas induced the lemma-like sterile lemmas (Jeon *et al.*, 2000; Wang *et al.*, 2017). Additionally, another class of genes, including *FRIZZY PANICLE* (*FZP*), *MULTI-FLORET SPIKELET1* (*MFS1*), *OsINDETERMINATE SPIKELET1* (*OsIDS1*), and *SUPERNUMERARY BRACT* (*SNB*) are also required to regulate the fate of glumes. Among these, loss-of-function of *SNB* and *FZP* induced extra rudimentary glumes with a loss of sterile lemmas on the corresponding rachilla position (Lee *et al.*, 2007; Komatsu *et al.*, 2003; Yi *et al.*, 2005; Bai *et al.*, 2017). Mutated *MFS1* and *OsIDS1* caused the transformation of sterile lemmas into rudimentary glumes (Ren *et al.*, 2013; Lee and An, 2012). These findings suggest that different genes are involved in the regulation of the fate of glumes, and the lemma, rudimentary glume, and sterile lemma are homologous organs. However, the identities and origins of glumes, and the relationship between the rudimentary glume, lemma, and sterile lemma are not yet fully understood in grass.

In the study, we identified a new weak mutant allele of *FZP*, a mutant we named *fzp-12*. In this mutant, *fzp-12* is mutated in a different genetic background and at a different site compared with the reported *fzp* mutants. In the previous studies, the *fzp* mutant failed to produce normal spikelets and grains and instead produced supernumerary rudimentary glumes (Bai *et al.*, 2016; Komatsu *et al.*, 2003). Interestingly, the *fzp-12* mutant showed different phenotypes, including small grains and degraded sterile lemmas. Therefore, we particularly investigated the functions of *FZP* in grain size and sterile lemma identity. Our results revealed that *FZP* regulates grain size by mediating cell proliferation and expansion. Our findings, along with data on the expression of *DL* and *G1* in sterile lemmas, also strongly support the hypothesis that the lemma, rudimentary glume, and sterile lemma are homologous organs. As such, our study provides a new function of *FZP*.

## Materials and methods

### Plant materials

The *fzp-12* mutant was obtained from an M_2_ population from the *japonica* cultivar Zhonghua 11 (ZH11) with ethylmethane sulfonate treatment. ZH11 was regarded as the wild type in this study. We crossed the *fzp-12* mutant with the cultivar Nan Jing 6 (NJ6, *indica*) to generate the F_2_ segregation population for gene mapping. Genetic analysis revealed that the *fzp-12* mutant trait was determined by a recessive single gene (Supplemental Table 1). All plants used for morphological and genetic analysis were cultivated in the paddies under natural growing conditions at the China National Rice Research Institute (Hangzhou, Zhejiang Province) and (Lingshui, Hainan Island), China.

### Microscopy analysis

For paraffin section observation, fresh spikelets from the wild type, *fzp-12* mutant, and *FZP* over-expressing plants were placed in 70% formalin-acetic acid-alcohol (FAA) mixed solution, then samples were treated and stained as described by Ren *et al*. (2016). Next, we observed the stained sections with a microscope (NIKON ECLIPSE 90i). Meantime, for scanning electron microscopy (SEM) analysis, young and fresh spikelets and panicles were examined using a scanning electron microscope with a −30 °C cooled stage (HITACHI S-3500).

### Map-based cloning and genetic transformation

To identify the mutated gene, the *fzp-12* mutant was crossed with the NJ6, and 1189 F_2_ recessive plants with the mutant trait were used for mapping. The mutated site of the candidate gene was identified by sequencing analysis. To test whether *LOC_Os07g47330/FZP* was responsible for the mutant phenotypes, we constructed complementation vectors. A genomic DNA fragment covering a 2025-bp promoter and a 1251-bp downstream sequences was cloned into the binary vector pCAMBIA1301 (*FZP*-COM). The 957-bp full-length coding region of *FZP* was inserted into the expression vector 35S-1301 with the 35S promoter (Ren *et al.*, 2016) to generate the 35S-*FZP* over-expression construct. Then, the *FZP*-COM and 35S-*FZP* plasmids were introduced into *Agrobacterium tumefaciens* and used to infect *fzp-12* calli. The 35S-*FZP* vectors was introduced into *A. tumefaciens* and used to infect ZH11 calli. The primer sequences in Supplemental Table 2.

### CRISPR-Cas9 targeting of *FZP*

For the CRISPR-Cas9 assay, we selected the specific candidate site in *FZP*. The CRISPR/Cas9 vector was constructed using the target sequence (GAGATACGCGACCCGACCACCA) to create the Cas9-*FZP* construct. The construct was introduced into *A. tumefaciens* and used to infect ZH11 calli.

### RNA isolation and quantitative reverse transcription PCR test

Total RNA was extracted and further purified following the related kit instructions (Axygen). First strand cDNA was synthesized using the ReverTra Ace quantitative PCR RT Master Mix Kit (Toyobo) using 2 µg purified RNA in a 20 µl reaction volume. The quantitative qPCR was performed as described by Ren *et al*. (2016). Three biological replicates were performed and rice *Actin* was used as an endogenous control. Primers used for qPCR analysis in Supplemental Table 2.

### *In situ* hybridization

Fresh young panicles from the wild type, *fzp-12* mutant, and *FZP* over-expressing plants were placed in 70% FAA mixed solution (RNase-free), dehydrated through ethanol and xylene, and then embedded in paraffin (Sigma). Hybridization and immunological treatment of the sections were performed according to the methods in Sang *et al.*, 2012. All probes were prepared using the same method and labeled with a RNA DIG Label (Roche). The primer sequences in Supplemental Table 2.

### Promoter activity analysis

The 2033-bp promoter being located upstream of the coding frame was fused to the *GUS* (ß-glucuronidase) reporter gene and inserted into pCAMBIA1391Z (pro*FZP*-GUS). The pro*FZP*-GUS plasmid was introduced into EHA105 and used to infect ZH11. GUS activity in the transgenic plants was detected as described by Rao *et al.* (2015). Primers in Supplemental Table 2.

### Subcellular localization of FZP protein

The coding frame of *FZP* was amplified from ZH11 and cloned into the 35S-GFP (S65T)-NOS (pCA1301) and 35S-YFP (S65T)-NOS (pCA1301) vectors to create the FZP-GFP and FZP-YFP recombinant vectors according to the In-Fusion Cloning Kit instructions (Takara). Then, the vectors of GFP and FZP-GFP, and the vectors of YFP and FZP-YFP were transformed into tobacco (*Nicotiana tabacum*) and rice protoplasts, and expressed, respectively (Ren *et al.*, 2016). The samples were observed using a confocal microscope (OLYMPUS IX71). The primer sequences in Supplemental Table 2.

### Yeast assay

The coding frame of *FZP* was amplified from ZH11. The coding frame of *GS2* was amplified from ZH11 and used as the positive control (Hu *et al.*, 2015). The two target fragments were inserted into the pGBKT7 vector to fuse with the GAL4 DNA-binding domain. All plasmids were transformed into yeast strain AH109 and the clones were selected on SD/–Trp plates. Then, the activation ability was tested on selective medium plates without histidine, tryptophan, and adenine (Ren *et al.*, 2016).

### Transcriptional activity analysis

Using the dual luciferase reporter assay system, we analyzed the transcriptional activity of FZP in rice protoplasts, and the DLR assay system was used with a GloMax 20-20 luminometer (Promega) to measure the relative luciferase activity (Wu *et al.*, 2013). The primers in Supplemental Table 2.

### Electrophoretic mobility shift assay

To produce the recombinant protein of HIS-FZP, the coding region of *FZP* was cloned into the pET-SUMO vector and expressed in the *E. coli* DE3 strain. The HIS-FZP recombinant proteins were purified as reported previously (Wang *et al.*, 2013). All probes containing three repeat motifs were synthesized by TSING KE (Hangzhou) and labeled with biotin at the 5’ end for the electrophoretic mobility shift assay (EMSA). One µg of probes and different concentrations of FZP recombinant protein were mixed with binding buffer in a total volume of 15 µl. EMSAs were conducted according to the instructions of the LightShift Chemiluminescent EMSA Kit (Thermo). The primers and probe sequences in Supplemental Table 2.

## Results

### The *fzp-12* mutant displays defects in the sterile lemma identity

**Fig. 1.**
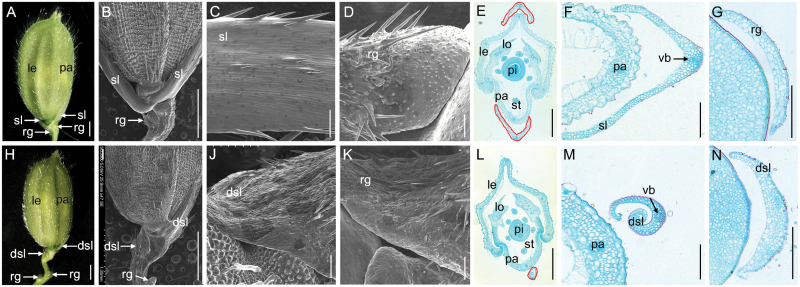
Phenotypes observation of spikelets in the wild type and the *fzp-12* mutant. A, wild type spikelet. B-D, scanning electronic microscope analysis of wild type spikelet. C, epidermal surface of the sterile lemma. D, epidermal surface of the rudimentary glume. E-G, histological analysis of wild type spikelet. F, histological analysis of the sterile lemma. G, histological analysis of the rudimentary glume. H, *fzp-12* spikelet. I-K, scanning electronic microscope analysis of *fzp-12* spikelet. J, epidermal surface of the sterile lemma. K, epidermal surface of the rudimentary glume. L-N, histological analysis of *fzp-12* spikelet. M, histological analysis of the sterile lemma. N, histological analysis of the sterile lemma. le, lemma; pa, palea; lo, lodicule; st, stamen; pi, pistil; sl, sterile lemma; dsl, degenerated sterile lemma; rg, rudimentary glume; vb, vascular bundle. Regions surrounded by red lines indicate the sterile lemma in E. Region surrounded by red line indicates the degenerated sterile lemma in L. Bars=1000 um in A, B, G, H; 100 um in C-F and I-L.

The rice spikelet comprises one terminal floret and two pairs of vestigial glumes (sterile lemmas and rudimentary glumes) (Fig. 1A); these develop from the spikelet meristem. The sterile lemmas are present on the rachilla in an alternate phyllotaxy and are formed between the terminal floret and the rudimentary glumes which occur below the sterile lemmas (Fig. 1A, B).

In wild-type plants, the sterile lemma of the spikelet differs from the rudimentary glume in size and structure (Fig. 1A–G). The sterile lemmas are larger and about 2 mm long, and the rudimentary glumes are about 0.5 mm long. Close examination revealed that in the *fzp-12* mutant, the reduced sterile lemma formed at the expense of the normal sterile lemma in the corresponding position (Fig. 1A, B, H, I). The *fzp-12* mutant produced smaller sterile lemmas that showed no visible differences from the rudimentary glumes of the *fzp-12* mutant or the wild type (Fig. 1A, B, H–K). To address the effect of the *fzp-12* mutation in detail, we investigated the cellular morphology of the sterile lemmas between the *fzp-12* mutant and the wild type. We found one vascular bundle in the wild-type sterile lemma, and the wild-type epidermis was smooth with regularly arranged cells with rare trichomes on the abaxial surface (Fig. 1C–F). No vascular bundles were found in the wild-type rudimentary glumes, and the epidermis was rough and developed irregularly arranged cells and had lots of trichomes and small protrusions on its surface (Fig. 1D, G). In contrast, the *fzp-12* mutant sterile lemmas were reduced to various degrees and even resembled the rudimentary glumes of the wild type or *fzp-12* mutant in size (Fig. 1H–N). Abundant trichomes and protrusions were observed on the epidermis of the reduced sterile lemmas (Fig. 1J), which were highly similar to the rudimentary glume (Fig. 1D). Also, the regularly arranged and smooth cells were found in the distal and marginal regions in the sterile lemmas of the *fzp-12* mutant (Fig. 1J), which were similar to those of the sterile lemma of the wild type. No homeotic transformations were observed in other spikelet organs in the *fzp-12* mutant (Fig. 1E, L). These results suggested that the *fzp-12* mutation specifically influences the sterile lemma, and the reduced sterile lemmas in the *fzp-12* mutant were converted to rudimentary glume-like organs that had the identities of both normal rudimentary glumes and sterile lemmas.

### Comparison of the early spikelet development between the wild type and *fzp-12* mutant

**Fig. 2.**
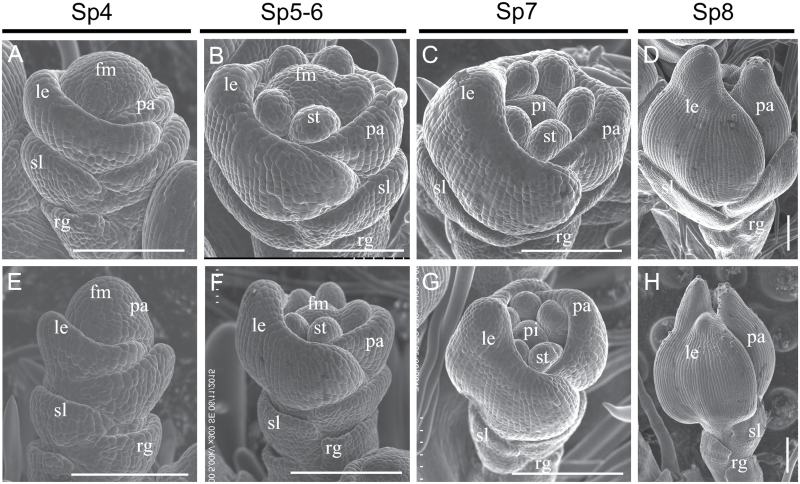
Developmental patterns of early spikelets in the wild type and the *fzp-12* mutant. A-D, wild type spikelet. E-H, *fzp-12* spikelet. fm, floral meristem; le, lemma; pa, palea; st, stamen; pi, pistil; sl, sterile lemma; rg, rudimentary glume; fm, floral meristem. Bars=100 μm.

To elucidate how defective spikelet development progressed in the *fzp-12* mutant, we monitored early developmental stages of young panicles by SEM (Fig. 2A–H; Fig. S1A–C). At the spikelet 4 stage (Sp4) of the wild type, the palea and lemma primordia were developing, and the wild-type sterile lemma was longer than the rudimentary glume (Fig. 2A). In the *fzp-12* mutant, the sterile lemma was similar to the rudimentary glume in morphology and size (Fig. 2E). In the wild type during Sp5-Sp6 stages, we observed stamen primordia, cessation of growth of the rudimentary glume, and continued growth of the sterile lemma (Fig. 2B). In the *fzp-12* mutant, the morphologies of the rudimentary glume and sterile lemma were not distinguishable from each other (Fig. 2F). During the Sp7 to Sp8 stage (when the pistil primordia are forming), the wild-type sterile lemma was much larger than the wild-type rudimentary glume (Fig. 2C, D, Fig. S1A–C). At these stages in the *fzp-12* mutant, the sterile lemma remained its original size, resembling the rudimentary glume (Fig. 2G–H, Fig. S1A–C).

**Fig. 3.**
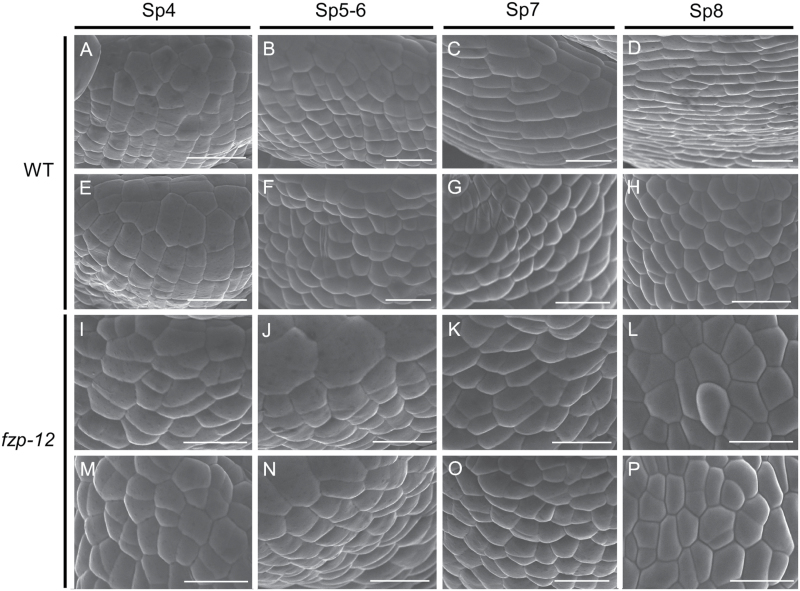
Development of sterile lemma and rudimentary glume in the wild type and *fzp-12* spikelets at early stages. A-D, epidermal surface of sterile lemma in the wild type. E-H, epidermal surface of rudimentary glume in the wild type. I-L, epidermal surface of sterile lemma in the *fzp-12* mutant. M-P, epidermal surface of rudimentary glume in the *fzp-12* mutant. Bars=10 μm.

Further observations of the base of the spikelet showed that the identity of epidermal cells of the sterile lemma in the *fzp-12* mutant is determined at the early stages (Fig. 3A–P). The surface of the sterile lemmas of the *fzp-12* mutant and the wild type were not distinguishable from each other before the stamen initiation (Fig. 3A, B; I, J). In the wild type after the stamen primordia were formed, the epidermal cells in the sterile lemmas started to elongate and formed an orderly arrangement (Fig. 3C, D). Moreover, the epidermal cells of rudimentary glume maintained their size in the wild type (Fig. 3E–H), whereas the epidermal cells in the reduced sterile lemmas of the *fzp-12* mutant became more similar to the cells of the rudimentary glume than the cells of the normal sterile lemma (Fig. 3K–P). These results implied that the identity of sterile lemma was altered in the *fzp-12* mutant, and the abnormal sterile lemma showed a similar developmental pattern with that of the wild-type rudimentary glume.

### The *fzp-12* mutant produces small grains

**Fig. 4.**
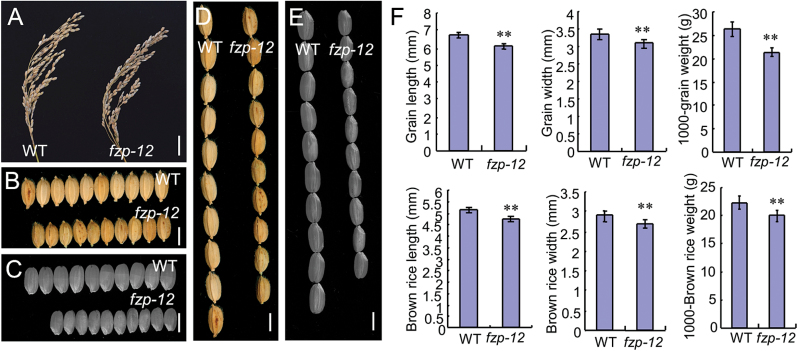
*FZP* affects grain size. A, panicle of the wild type and *fzp-12* mutant. B, mature grains of the wild type and *fzp-12* mutant. C, brown rice of the wild type and *fzp-12* mutant. D, mature grains of the wild type and *fzp-12* mutant. E, brown rice of the wild type and *fzp-12* mutant. F, length, width, and weight of mature grains and brown rice in the wild type and *fzp-12* mutant. Bars=2 cm in A; 5 mm in B-E. **Significant difference at *P*<0.01 compared with the wild type by Student’s *t*-test.

The *fzp-12* mutant had obviously smaller grains than the wild type, and no obvious differences of grain number per panicle and seed setting rate were found in the wild type and *fzp-12* mutant (Fig. 4A–F, Fig. S1D). Observations of early spikelet development showed that these spikelets were compressed and became dwarf throughout the early spikelet development stages (Fig. 2A–H), consistent with the smaller *fzp-12* grains. At maturity, the widths of the grains and brown rice from the *fzp-12* mutant were reduced compared with those of the wild type (Fig. 4B, C, F). The average widths of the grains and brown rice from the wild type were 3.3 and 3.0 mm, respectively (Fig. 4F), while the average widths of the grains and brown rice from the *fzp-12* mutant were 2.9 and 2.7 mm, respectively (Fig. 4F). Also, the *fzp-12* mutant grains and brown rice were shorter in comparison with those of the wild type (Fig. 4D–F). The average lengths of the grains and brown rice were 6.7 and 5.2 mm in the wild type, respectively (Fig. 4F), while the grains and brown rice from the *fzp-12* mutant averaged 6.1 and 4.7 mm long, respectively (Fig. 4F). Additionally, the weights of 1000-grain and 1000 brown rice in the *fzp-12* mutant were considerably decreased compared to the wild type (Fig. 4F). These results led to significantly decreased grain yield per plant in the *fzp-12* mutant compared with the wild type (Fig. S1D). Together, these findings revealed that *FZP* affects grain length and width as well as grain yield.

### Molecular cloning of *FZP*

**Fig. 5.**
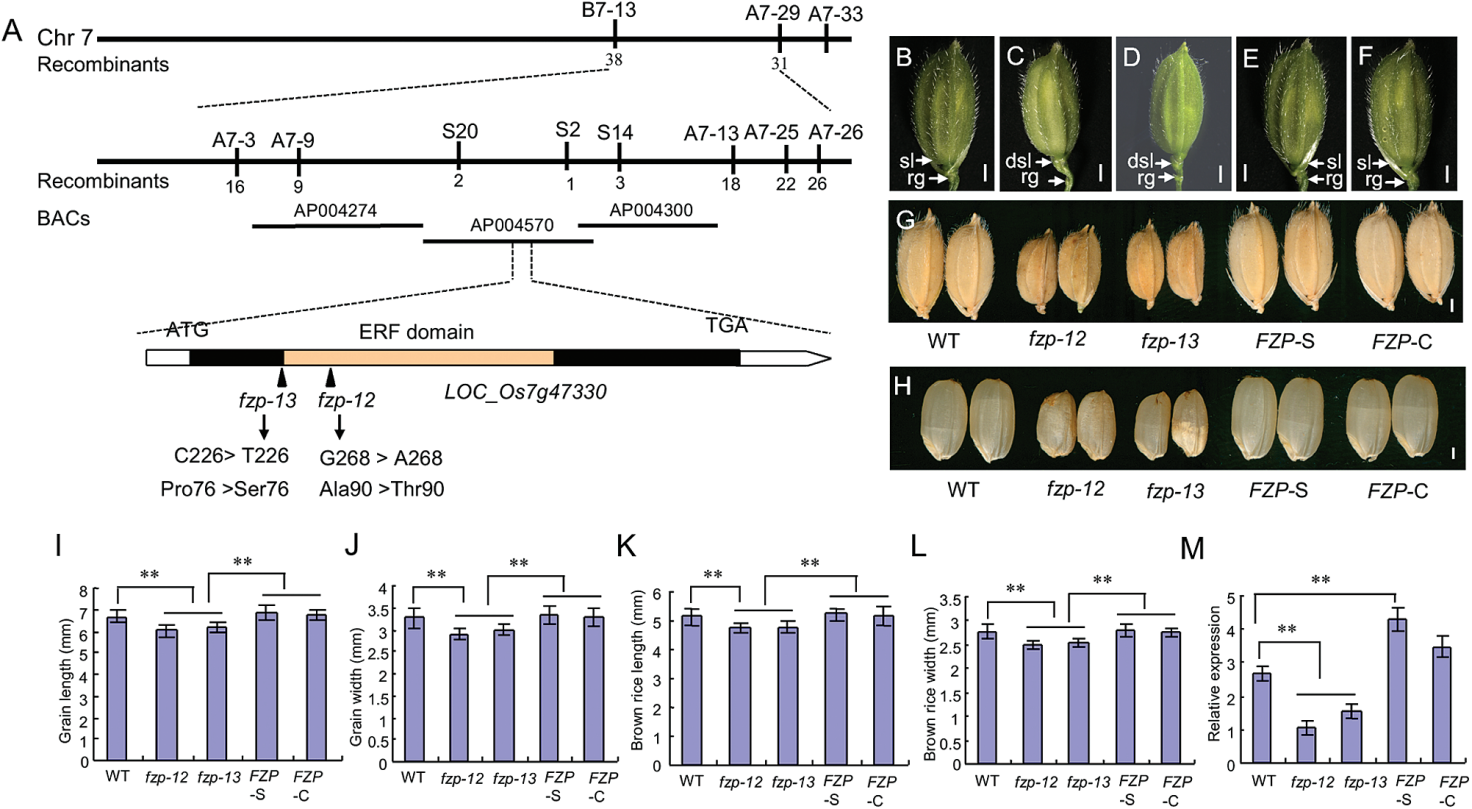
Map-based cloning of the mutated gene. A, map position of *fzp-12* locus. B, wild type spikelet. C, *fzp-12* spikelet. D, *fzp-13* spikelet. E, defective spikelets of *fzp-12* mutant were rescued in the 35S::*FZP*–GFP complementation plants. F, defective spikelets of *fzp-12* mutant were rescued in the genomic complementation plants. G, mature grains. *fzp-13* mutant produced smaller grains, resembling those of *fzp-12* mutant. And small grains of *fzp-12* mutant were rescued in 35S::*FZP*–GFP complementation plants and genomic complementation plants. H, brown rice. *fzp-13* mutant produced smaller brown rice grains, resembling those of *fzp-12* mutant. And small brown rice grains of *fzp-12* mutant were rescued in 35S::*FZP*–GFP complementation plants and genomic complementation plants. I, grain length. J, grain width. K, brown rice length. L, brown rice width. M, expression levels of *FZP* in the wild type, *fzp-12* mutant, *fzp-13* mutant, 35S::*FZP*–GFP complementation plants and genomic complementation plants. *fzp-13* indicates the Cas9 knock-out plant. *FZP*-S indicates the 35S::*FZP*–GFP complementation plants. *FZP*-C indicates the genomic complementation plants. dsl, degenerated sterile lemma; sl, sterile lemma; rg, rudimentary glume. Bars=1 mm in B-H. Error bars indicate SD. **Significant difference at *P*<0.01 compared with the wild type by Student’s *t*-test.

To isolate the mutated gene that determined the mutant phenotypes, we performed a cross between the *fzp-12* mutant and NJ6, and all F_1_ plants showed the wild-type phenotype. In the F_2_ population, 3612 individuals exhibited the wild-type phenotype and 1189 plants exhibited the *fzp-12* mutant phenotype, which fits the Mendelian segregation ratio (3:1) of a single recessive nuclear gene (Supplemental Table 1). Sixty individuals showing the *fzp-12* phenotype were selected from the F_2_ population, and the mutation was preliminarily located between the simple-sequence repeat (SSR) markers B7-13 and A7-29 on chromosome 7 (Fig. 5A). Next, we used 36 SSR/SNP markers and 1129 recessive plants for fine mapping. The *fzp-12* locus was delimited between the markers S2 and S20, a 66-kb region, which contained 7 open reading frames (ORFs) (Fig. 5A). By sequencing all predicted genes within this region, we found a single-nucleotide mutation from G to A within an ERF transcription factor (*LOC_Os07g47330/FZP*), which triggered an amino acid substitution of Ala-90 to Thr-90 in the *fzp-12* mutant (Fig. 5A; Fig. S2).

To confirm whether *LOC_Os07g47330* is causally linked to the *fzp-12* mutant phenotypes, two plasmids, one contained the *LOC_Os07g47330* wild-type genomic fragment and the other containing the wild-type cDNA driven by a 35S promoter (35S::FZP–GFP), were introduced into the *fzp-12* calli. We obtained 28 and 20 transgenic lines from each transformation, respectively. Among which, 16 and 12 lines, respectively, harbored the transgene, and were resistant to hygromycin, and showed the wild-type phenotypes, which revealed that the *fzp-12* phenotypes were completely rescued (Fig. 5B, C; E–M). Furthermore, we used the CRISPR/Cas9 gene editing to knock out *FZP* in the ZH11 wild-type plants. Sequencing analysis exhibited that the homozygous knocked out *fzp-13* mutant had one single base mutation from C to T, resulting in an amino acid substitution of Pro-76 to Ser-76 (Figs 5A; S2). In the *fzp-13* mutant, the expression of *FZP* was greatly reduced and this plant produced smaller grains and degenerated sterile lemmas (Fig. 5B–D; G–M), resembling the *fzp-12* phenotypes. Additionally, we also found the knocked out *fzp-14* mutant in the transgenic plants, and this mutant produced many branches that replaced the spikelets, resembling the phenotypes of previous reported *fzp* mutants. Sequencing analysis showed that the *fzp-14* mutant showed one single base C deletion, resulting in a premature translation stop (Fig. S3). Together, these data confirmed that *LOC_Os07g47330*/*FZP* is the target gene, the mutated *FZP* is responsible for the mutant phenotypes.

### *FZP* expression pattern and subcellular localization of its encoding protein

To determine the *FZP* expression pattern, we detected its expression in the *fzp-12* mutant and wild type. The qPCR analysis showed that *FZP* was mainly expressed in young panicles, with higher levels in panicles shorter than 2 cm (Fig. S4A). No notable expression was found in the other tissues examined including roots, stems, leaves, and sheaths (Fig. S4A). However, *FZP* showed lower transcript levels in the *fzp-12* mutant compared to the wild type (Fig. S4A). We fused the reporter gene (GUS gene) with the *FZP* promoter (pro*FZP*::GUS), and transformed the recombinant plasmid into ZH11. GUS activity from pro*FZP*-GUS was only found in the spikelets (Fig. S4B–F). Furthermore, Cross-section of the GUS stained spikelets displayed that the *FZP* promoter was specifically active in the outer spikelet organs including the sterile lemma, lemma and palea (Fig. S4G–I). And, no obvious GUS activity was observed in inner floral organs including the lodicule, stamen, and pistil (Fig. S4I). The expression pattern of *FZP* was different from the reported *in situ* hybridization expression pattern (Komatsu *et al.*, 2003), and was consistent with its function in influencing spikelet development and grain size.

The FZPORF-GFP fusion protein and GFP alone, and the FZPORF-YFP fusion protein and YFP alone were transiently expressed in rice protoplasts and tobacco (*Nicotiana tabacum*) epidermal cells, respectively (Fig. S5). In cells expressing GFP or YFP only, the green and yellow fluorescence signals were visible throughout the cell (Fig. S5A–C; G–I). And, green fluorescence and yellow fluorescence were observed in the nuclei of rice protoplasts and tobacco epidermal cells containing the FZPORF-GFP and FZPORF-YFP fusion proteins (Fig. S5D–F; J–L). These results indicated that *FZP* encodes a nuclear-localized protein that may function as a transcription factor.

### Transcriptional activity analysis of FZP

**Fig. 6.**
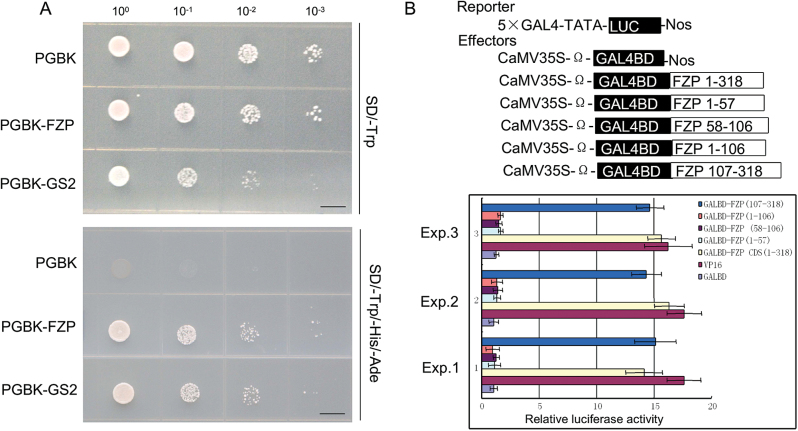
Transcriptional activity analysis of FZP protein. A, transcriptional activation assay in yeast cell. B, relative luciferase activities of rice protoplasts. PGBK, PGBK-FZP, and PGBK-GS2 represent the empty PGBK vector, the pGBKT7 vector fused with the coding frame of FZP, and the pGBKT7 vector fused with the coding frame of GS2. Bars=1 cm.

To explore the transcriptional activity of FZP, we fused the coding regions of *FZP* and the known transcriptional activator *GS2* to the DNA-binding domain (BD) of yeast GAL4. The BD-*GS2* construct and empty pGBKT7 vector were regarded as positive and negative controls, respectively (Hu *et al.*, 2015). The transformed yeast cells harboring BD-*FZP* and BD-*GS2* grew on the medium without histidine, tryptophan, and adenine, whereas the yeast cells harboring the pGBKT7 empty vector did not survive on the medium without histidine, tryptophan, and adenine (Fig. 6A), suggesting that FZP has transcriptional activation activity.

Next, we used a dual luciferase (LUC) reporter to further investigate the transcriptional activity, and examined the LUC activity in rice protoplasts. As in the yeast assays described above, the coding frame of the *FZP* cDNA was fused to the GAL4 DNA-binding domain (BD), but it was driven by the constitutive 35S promoter from *Cauliflower mosaic virus* (BD-FZP). The *LUC* gene that has five copies of binding sites was used for GAL4. VP16, a transcriptional activator, was used as a positive control and GAL4-BD was regarded as a negative control (Ren *et al.*, 2016). The VP16, BD-FZP, and GAL4-BD effectors were transiently expressed in rice protoplasts. FZP and VP16 showed higher luciferase activity than that of GAL4-BD (Fig. 6B). These findings further demonstrated that FZP acts as a transcriptional activator and has transcriptional activation activity, implying that FZP may be involved in transcriptional regulation.

Further, we investigated the transcriptional activation activity of a set of truncated FZP proteins. The result revealed that the N-terminal regions including 1–57, 58–106 and 1–106 had no transcriptional activation activity, whereas the C-terminal region 107–318 conferred the same transactivation activity as FZP, suggesting that the C terminal region (107–318) is indispensable for the transcriptional activation activity (Fig. 6B).

### Expression patterns of marker genes in the spikelet

**Fig. 7.**
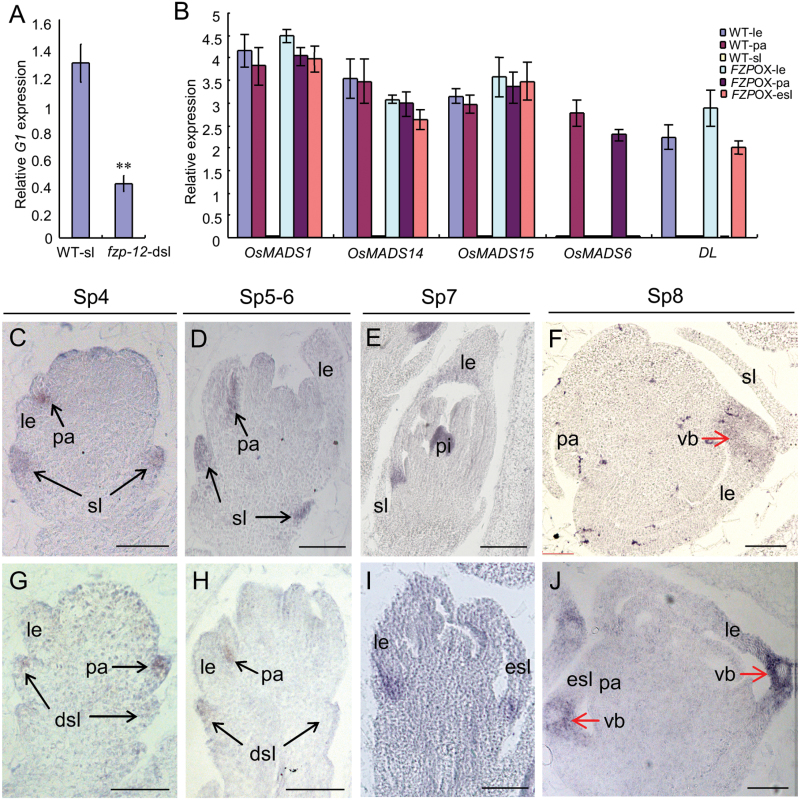
Expression of floral organ identity genes in the spikelets of the wild type, *fzp-12* mutant and *FZP* over-expressing plant. A, qPCR analysis of *G1* gene in the wild type and *fzp-12* mutant. B, qPCR analysis of *DL*, *OsMADS15*, *OsMADS14*, *OsMADS6*, and *OsMADS1* genes in the wild type and *FZP* over-expressing plant. C-D, *G1* expression in the wild-type. E-F, *DL* expression in the wild-type. G-H, *G1* expression in the *fzp-12* mutant. I-J, *DL* expression in the *FZP* over-expressing plant. dsl, degenerated sterile lemma; esl, enlarged sterile lemma; sl, sterile lemma; le, lemma; pa, palea; pi, pistil; vb, vascular bundle. Bars=50 μm. Error bars indicate SD. **Significant difference at *P*<0.01 compared with the wild type by Student’s *t*-test.

We used qRT-PCR to examine the expression of several genes that specify the sterile lemma or hull (lemma and palea). The expression of the sterile lemma identity gene, *G1*, was significantly decreased in the *fzp-12* reduced sterile lemmas compared with that of the wild type (Fig. 7A), suggesting that the sterile lemma identity was affected in the *fzp-12* mutant. In the wild type, the transcripts of the hull marker genes *OsMADS15*, *OsMADS14,* and *OsMADS1*, were detected in the palea and lemma, and the palea identity gene, *OsMADS6*, and the lemma identity gene, *DL*, were primarily expressed in the palea and lemma, respectively (Fig. 7B). In the *FZP* over-expressing plants, the sterile lemma was enlarged and had similar vascular bundles, cell layers, inward hook-like structures, and epidermal cells, including trichomes and protrusions, resembling those of the lemma in the wild type (Fig. S6A–H). Abundant transcripts of *DL*, *OsMADS15*, *OsMADS14*, and *OsMADS1* were found, but no *OsMADS6* transcript was observed in the elongated sterile lemmas (Fig. 7B). Meantime, the expressions of *DL*, *OsMADS6*, *OsMADS15*, *OsMADS14*, and *OsMADS1* were not changed in the *fzp-12* mutant (Fig. S7).

Next, we investigated the expression of *G1* and *DL* by *in situ* hybridization (Fig. 7C–J). Strong *G1* expression was detected in sterile lemmas at Sp4-Sp6 stages in the wild type (Fig. 7C, D), but *G1* signals were faint in the sterile lemma primordia at these stages in the *fzp-12* mutant (Fig. 7G, H), suggesting that the degenerated sterile lemmas were derived from the normal sterile lemmas and partly retained the sterile lemma identity. In wild-type flowers, a strong *DL* signal was visible in the lemma at the Sp7-Sp8 stages (Fig. 7E, F), whereas in the *FZP* over-expressing plants, *DL* signals were observed in the elongated sterile lemmas and the original lemma (Fig. 7I, J), indicating that *FZP* over-expression induced a homologous change of the sterile lemma to the lemma. Thus, these results revealed that *FZP* indeed regulated the sterile lemma identity.

### *FZP* influences cell proliferation and expansion in spikelet hulls

**Fig. 8.**
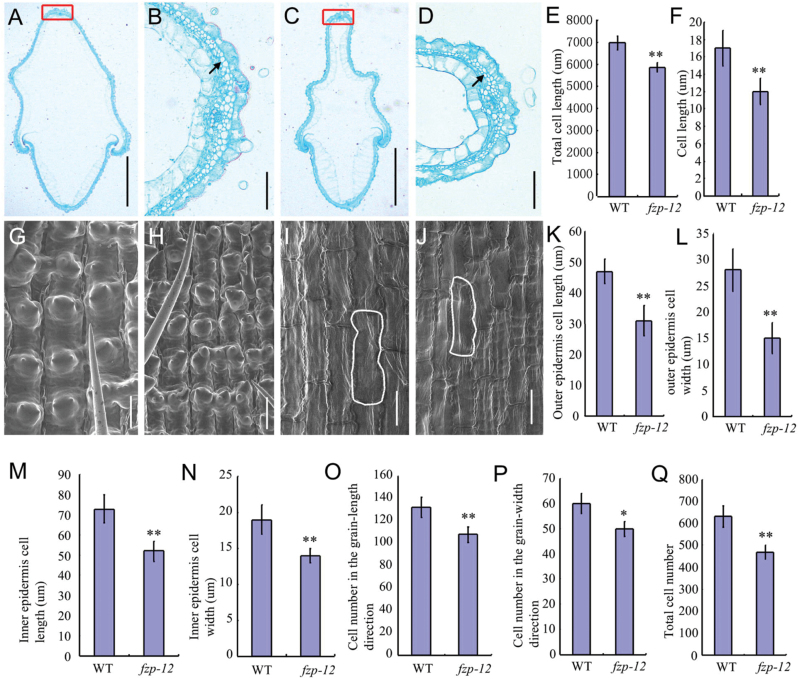
*FZP* influences cell proliferation and expansion in spikelet hulls. A, cross-sections of spikelet hulls in the wild type. B, magnified views of the box in A. C, cross-sections of spikelet hulls in the *fzp-12* mutant. D, magnified views of the box in C. E, total cell length in the outer parenchymal cell layers of spikelet hulls. F, cell length in the outer parenchymal cell layers of spikelet hulls. G, SEM analysis of the outer surface in the wild type. H, SEM analysis of the outer surface in the *fzp-12* mutant. I, SEM analysis of the inner surface in the wild type. J, SEM analysis of the inner surface in the *fzp-12* mutant. K, outer epidermis cell length. L, outer epidermis cell width. M, inner epidermis cell length. N, inner epidermis cell width. O, cell number in the grain-length direction. P, cell number in the grain-width direction. Q, total cell number of outer parenchymal cell layers of spikelet hulls. Arrows indicate the parenchymal cell. Bars=1000 um in A and C; 100 um in B and D; 50 um in G and J. **Significant difference at *P*<0.01 compared with the wild type by Student’s *t*-test; *Significant difference at *P*<0.05 compared with the wild type by Student’s *t*-test.

In rice, the spikelet hull restricts grain growth, thereby determining grain size (Duan *et al.*, 2016). During organogenesis, cell proliferation and expansion coordinately regulate the organ growth (Jiang *et al.*, 2012). To reveal the cellular basis underlying the change in grain size, we investigated cell size and number in the hulls of the wild type and the *fzp-12* spikelet by SEM and paraffin sectioning (Fig. 8A–Q). Cross-sections of central region of the spikelet hull revealed that the parenchymal cell layer in the *fzp-12* mutant hull was shorter and the hull contained smaller and fewer cells than the same cell layer in the wild type (Fig. 8A–D; E, F; Q). Similarly, there were fewer outer epidermal cells in the grain-width direction than in the wild type (Fig. 8P). The *fzp-12* spikelet hulls had narrower and shorter cells in the outer epidermis than those of the wild-type spikelet hulls (Fig. 8G, H; K, L). Similarly, the *fzp-12* mutant spikelet hulls had short, narrow cells in the inner epidermis compared to those of the wild type (Fig. 8I, J; M, N). We measured the number of outer epidermal cells along the longitudinal grain-length direction in the wild type and the *fzp-12* mutant. The total cell number along the longitudinal grain-length direction was significantly reduced in the *fzp-12* mutant compared with that of the wild type (Fig. 8O). These results suggested that the small-grain phenotype of the *fzp-12* mutant mainly results from changes in cell size and number in the spikelet hull.

Several grain-size genes regulate cell proliferation and expansion. *SPL13/GWL7*, *GL7/GW7/SLG7, GS2, SRS5*, *PGL1*, *PGL2*, and *APG* influence cell expansion, and *GS3*, *GL3*, *GS5*, *GW2*, and *GW8* are involved in regulation of cell proliferation. To reveal how *FZP* regulates cell proliferation and expansion in spikelet hulls, we detected their expression levels in the wild-type and *fzp-12* mutant panicles. Compared with the wild type, the expression levels of *GWL7*, *SRS5*, *GL7, PGL1*, *PGL2*, *GS3*, *GS5*, and *GW8* were significantly reduced in *fzp-12* panicles (Fig. S8A), and the expression levels of *GS2, GL3*, *GW2*, *APG* in *fzp-12* panicles were similar between the *fzp-12* mutant and the wild type (Fig. S8A).

Furthermore, we investigated the expression of several genes that regulate cell cycle and cell expansion. Compared with the wild type, ten cell cycle-related genes, *CYCT1*, *CYCA2-1*, *CYKA2*, *CYCD3*, *CYCD20*, *H1*, *E2Fa*, *MAD2*, *MCM3*, and *MCM5*, showed significantly lower expression in the *fzp-12* mutant than in wild type (Fig. S8B). The expression levels of five cell expansion-related genes, *OsEXPA32*, *OsEXPA2*, *OsEXPA1*, *OsEXPB11*, and *OsEXPB9*, were also much lower in the *fzp-12* mutant compared with that of the wild type (Fig. S8B). Together, these results supported the role of *FZP* in the regulation of cell proliferation and expansion in spikelet hulls, and thereby in grain size.

### FZP interacts specifically with the GCC-box and DRE motifs

**Fig. 9.**
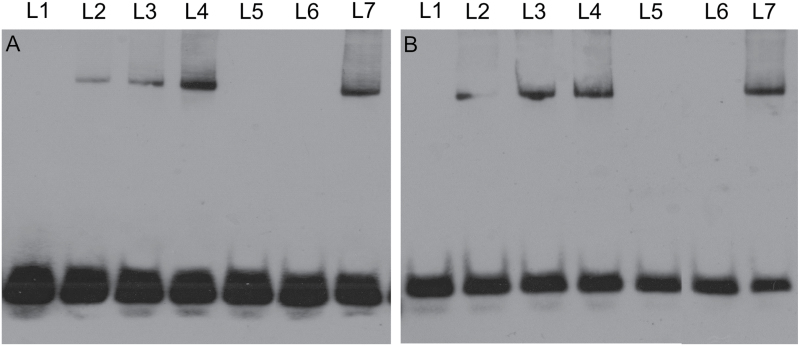
FZP protein interacts with the GCC-box and DRE motifs. A, FZP protein could bind to the normal GCC-box and failed to interact with mutated GCC-box (mGCC-box). B, FZP protein could bind to the normal DRE and failed to interact with mutated DRE (mDRE). L1, L2, L3 L4 L5, L6 and L7 in A indicate GCCbox, GCCbox+0.5 ug protein, GCCbox+1 ug protein, GCCbox+1.5 ug protein, mGCCbox+1.5 ug protein, GCCbox+1.5 ug protein+100×specific competitor, and GCCbox+1.5 ug protein+100×non-specific competitor, respectively. L1, L2, L3 L4 L5, L6 and L7 in in B indicate DRE, DRE+0.5 ug protein, DRE+1 ug protein, DRE+1.5 ug protein, mDRE+1.5 ug protein, DRE+1.5 ug protein+100×specific competitor, and DRE+1.5 ug protein+100×non-specific competitor, respectively. L, lane.

The GCC-box and dehydration-responsive element (DRE) motifs are recognized by ERF subfamily proteins, which belong to the AP2/ERF superfamily (Rong *et al.*, 2014). Therefore, ERF proteins can directly or indirectly activate the expression of downstream genes with the GCC-box or DRE motifs, thereby affecting plant development. In this study, *FZP* also encodes an ERF domain protein and belongs to the AP2/ERF superfamily. We investigated whether FZP binds to the GCC-box and DRE motifs, as well as to GCC-box and DRE motifs containing two point mutations, by an electrophoretic mobility shift assay (EMSA). The EMSA results showed that FZP could directly combine with the normal GCC-box and DRE DNA fragments, but it failed to combine with the mutated GCC-box and DRE DNA fragments (Fig. 9A, B). Additionally, different concentrations of FZP exhibited different binding abilities with the normal GCC-box and DRE DNA fragments (Fig. 9A, B). These results indicated that FZP interacts specifically with the GCC-box and DRE motifs in the promoters of related downstream genes, and may thereby directly regulate their expression.

## Discussion

### *FZP* plays an important role in determining grain size

Grain size is coordinately determined by grain length, width and thickness, which are controlled by several genetic factors in rice. However, our knowledge of the complicated genetic mechanisms by which rice grain size is controlled is still limited.

*FZP* encodes an ERF protein with transcriptional activation activity, and the *fzp-12* loss-of-function mutant showed short and narrow grains. Early spikelets of *fzp-12* mutant became dwarf, consistent with the smaller *fzp-12* grains. The knocked out *fzp-13* mutant also produced small grains that resembled those of the *fzp-12* mutant. These results suggest that *FZP* is an important regulator of grain size and shape in rice. Cell proliferation and cell expansion determine organ size (Jiang *et al.*, 2012). Our results revealed that *FZP* is required for cell proliferation and expansion in rice grains. The spikelet hulls of the *fzp-12* mutant had smaller and fewer cells than the wild-type spikelet hulls, and the transcript levels of several genes involved in cell proliferation and expansion were significantly reduced in the *fzp-12* mutant. These findings suggested that *FZP* influences grain size by modifying cell proliferation and expansion.

### *FZP* determines sterile lemma fate in rice

The sterile lemma and rudimentary glume of grasses are unique organs (Li *et al.*, 2009). In previous studies, the *fzp* mutants only produced supernumerary bract-like rudimentary glumes, and lacked normal florets and sterile lemmas (Komatsu *et al.*, 2003; Yi *et al.*, 2005). Rarely, the bract-like organs partly harbored the identity of the sterile lemma in the *fzp* mutant (Komatsu *et al.*, 2003). In our study, the *fzp-12* and knocked out *fzp-13* mutants produced the spikelets with one pair of reduced sterile lemmas, which resembled the rudimentary glume of the wild type and the bract-like organs of the reported *fzp* mutants in size. The sterile lemmas of the *fzp-12* and *fzp-13* mutants, and the bract-like organs of the reported *fzp* mutants exhibited similar protrusions and trichomes to those of the rudimentary glume of the wild type (Fig. 1; Komatsu *et al.*, 2003). These results indicated that these reduced glume-like organs or bract-like rudimentary glumes may be derived from the sterile lemmas. Next, we found no expression of *DL*, *OsMADS15*, *OsMADS14*, *OsMADS6*, and *OsMADS1* in sterile lemmas of the wild type and *fzp-12* mutant, suggesting that the sterile lemma of the *fzp-12* mutant does not have the lemma/palea identity. Compared with the wild type, *G1* signals were significantly decreased in the sterile lemmas of the *fzp-12* mutant, indicating that the sterile lemma identity is altered in the *fzp-12* mutant. The sterile lemma in the *FZP* over-expressing plants was enlarged and had a similar cellular structure, resembling the wild-type lemma. We detected abundant transcripts of *DL*, *OsMADS15*, *OsMADS14*, and *OsMADS1*, but no *OsMADS6* signals were found in the sterile lemmas of the *FZP* over-expressing plants. These findings indicated that the sterile lemma is converted to the lemma-like organ and had the lemma identity in these plants. Cross-section of the spikelets showed that GUS activity was obviously visible in the sterile lemma, which was consistent with our phenotypic observations (i.e. reduced sterile lemmas). Together with the strong expression of *FZP* in the sterile lemma, our results showed that *FZP* controls the identity of the sterile lemma, and is vital in the development of sterile lemma by repressing the development of the rudimentary glume.

Two hypotheses have been proposed on the evolution and origin of the sterile lemma (Ren *et al.*, 2013; Takeoka *et al.*, 1993). One hypothesis suggests that the spikelet of a putative ancestor of *Oryza* contained three florets: a terminal floret and two lower lateral florets. In time, the lateral florets lost all inner floral organs and subsequently degenerated into the lemma (Kellogg, 2009). The sterile lemma was regarded as the remnant of the morphologically modified lemma (Yoshida *et al.*, 2009). An alternative hypothesis proposes that the spikelet of a putative ancestor of *Oryza* only produced one floret, and the rudimentary glume and sterile lemma were reduced, bract-like organs. In the *dg1*, *g1*, *eg1*, *asp1*, and *osmads34* mutants, the sterile lemmas were elongated and transformed into lemma-like organs, which supports the first idea that the lemma and sterile lemma were homologous organs. In the *osids1* and *mfs1* mutants, the sterile lemma was not fully formed and acquired the identity of the rudimentary glume, thereby supporting the second hypothesis and the view that the rudimentary glume and sterile lemma were homologous organs. In our study, the sterile lemma of the *fzp-12* mutant was reduced and resembled the rudimentary glume, whereas the sterile lemma was elongated and converted to the lemma-like structure in the *FZP* over-expressing plants. In addition, the rudimentary glumes were also enlarged and had the identity of the sterile lemma or lemma in the *asp1*, *dg1*, and *osmads34* mutants (Yoshida *et al.*, 2012; Ren *et al.*, 2016; Yu *et al.*, 2017). The spikelet of most grass species contains florets and glume-like organs that are equivalent to the rudimentary glumes of *Oryza* spp., but lacks sterile lemma-like organs (Hong *et al.*, 2010; Yoshida *et al.*, 2009). In some grass species, the bract-like glume organ is similar in structure and size to the lemma (Yoshida *et al.*, 2009; Ren *et al.*, 2013), whereas in *Oryza* spp., this organ is severely reduced (Bommert *et al.*, 2005; Li *et al.*, 2009; Ren *et al.*, 2013). Together with these hypotheses and previous studies, our results strongly support the view that the lemma, rudimentary glume, and sterile lemma were homologous structures.

### A conserved function of AP2/ERF genes

**Fig. 10.**
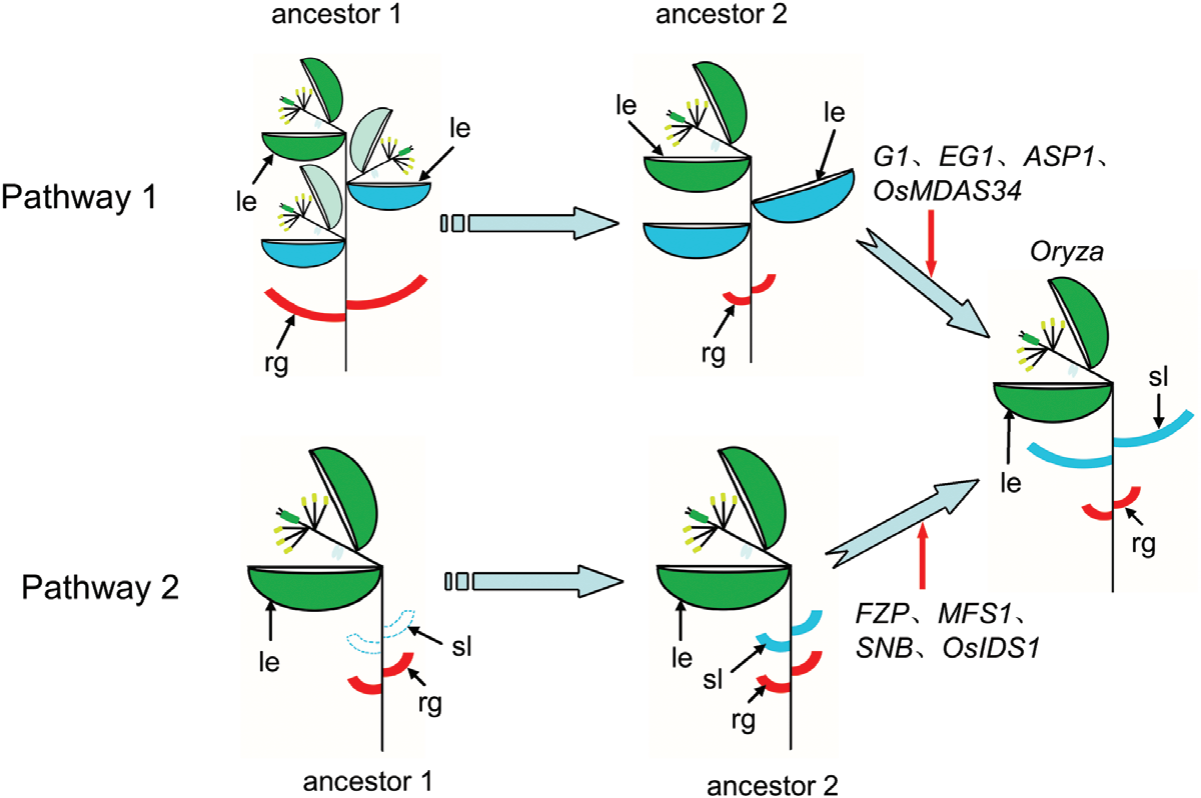
Hypothesized evolutionary pathway in spikelet architecture of *Oryza* (Yoshida *et al.*, 2009) and a conserved function of AP2/ERF genes. rg, rudimentary glume; sl, sterile lemma; le, lemma. The pathway 1 indicates the evolutionary process in which the sterile lemma is gradually reduced, and this hypothesized process has been shown (Yoshida *et al.*, 2009). The pathway 2 indicates the evolutionary process in which the sterile lemma starts to appear and elongate. The putative ancestor 1 of genus *Oryza* had three florets (one terminal floret and two lower lateral florets) and a pair of rudimentary glumes in a single spikelet in the pathway 1. The putative ancestor 2 of genus *Oryza* had one terminal floret, two lower lemma-like organs, and a pair of rudimentary glume in a single spikelet in the pathway 1. The putative ancestor 1 of genus *Oryza* had one terminal floret and a pair of rudimentary glume, and lacked the sterile lemma-like organ in a single spikelet in the pathway 2. The putative ancestor 2 of genus *Oryza* had one terminal floret and two rudimentary glume-like organs in a single spikelet in the pathway 2.

The plant-specific AP2/ERF gene family includes four subfamilies: ERF, AP2, DREB, and RAV (Sharoni *et al.*, 2011). Previous studies showed that *SNB* and *OsIDS1* encode an AP2 domain protein, and play a role in the specification of sterile lemma fate. *FZP* and *MFS1* belong to the ERF subfamilies and also determine the sterile lemma identity. The plants with mutations in *SNB* produced extra rudimentary glumes; however, no corresponding sterile lemmas were observed in the original position. Loss of function of *FZP*, *MFS1*, and *OsIDS1* caused the transformation of the sterile lemmas into rudimentary glumes. These AP2/ERF genes have a unique AP2 domain (Fig. S9), showed similar expression patterns and were strongly expressed in the sterile lemma. The other class of genes including *EG1*, *ASP1*, *G1*/*ELE1*, and *OsMADS34*, encode non-AP2/ERF domain proteins, and also affect the development of the sterile lemma. The sterile lemmas were elongated and had the lemma identity in *eg1*, *asp1*, *g1* and *osmads34* mutants. These findings suggest that the development of the sterile lemma is controlled by two different regulatory pathways. *EG1*, *ASP1*, *G1*/*ELE*, and *OsMADS34* determine the sterile lemma identity by preventing the homologous transformation of the sterile lemma into the lemma, whereas, the functions of the AP2/ERF genes *FZP*, *MFS1*, *OsIDS1*, and *SNB* are conserved in the regulation of the sterile lemma identity by mainly preventing the degeneration of the sterile lemma into the rudimentary glume. Interestingly, *O. grandiglumis* has a large sterile lemma that resembles the lemma, whereas the genus *Leersia* belongs to Oryzeae and lacks the sterile lemma. These results support the above-mentioned hypotheses, and suggest the existence of four putative ancestors of *Oryza* spikelet that lead to the different types of spikelets and two evolutionary pathways that determine the origin of sterile lemma (Fig. 10). In hypothesized pathway 1, the putative ancestor of the spikelet of genus *Oryza* contained three florets: two lateral florets and one terminal floret. Subsequently, the lateral florets degenerated into the lemmas, consistent with the previous idea (Yoshida *et al.*, 2009; Kellogg, 2009; Ren *et al.*, 2013), suggesting that *EG1*, *ASP1*, *G1*/*ELE*, and *OsMADS34* may be recruited to specify the sterile lemma by repressing the lemma identity during evolution (Fig. 10). In hypothesized pathway 2, the putative ancestor of the spikelet of genus *Oryza* had a terminal floret and a pair of rudimentary glumes, similar with the genus *Leersia*. During evolution, the spikelet developed the rudimentary glume-like sterile lemma, which subsequently were elongated, suggesting that the AP2/ERF genes (*FZP*, *MFS1*, *OsIDS1*, and *SNB*) may be recruited to specify the sterile lemma by suppressing the rudimentary glume identity (Fig. 10).

## Supplementary data

Supplementary data are available at *JXB* online.

Supplemental Figure 1. Investigation of early spikelets and agronomic traits in the wild type and *fzp-12* mutant.

Supplemental Figure 2. Protein sequence alignment of FZP, fzp-12, and fzp-13.

Supplemental Figure 3. *fzp-14* mutant and protein sequence alignment of FZP and fzp-14.

Supplemental Figure 4. Expression pattern of *FZP*.

Supplemental Figure 5. Subcellular localization of the FZP protein.

Supplemental Figure 6. Spikelets and *FZP* expression in the wild type and *FZP* over-expressing plants.

Supplemental Figure 7. Expression of floral organ identity genes in the wild type and *fzp-12* mutant.

Supplemental Figure 8. Expression levels of related genes in the wild type and *fzp-12* mutant.

Supplemental Figure 9. Protein sequence alignment of AP2/ERF genes.

Supplemental Table 1. Genetic analysis of the mutant phenotypes of *fzp-12*.

Supplemental Table 2. Primers used in the study.

## Supplementary Material

Supplementary Material
