# Supplementary material for: FZP determines grain size and sterile lemma fate in rice
Source: J Exp Bot. 2018 Jul 19;69(20):4853–66. doi: 10.1093/jxb/ery264 (PMC6137974; doi:10.1093/jxb/ery264)
Supplement: Supplementary Material [file ery264_suppl_supplementary_material.pdf]

## SUPPLEMENTAL FIGURES

### Supplemental Figure 1

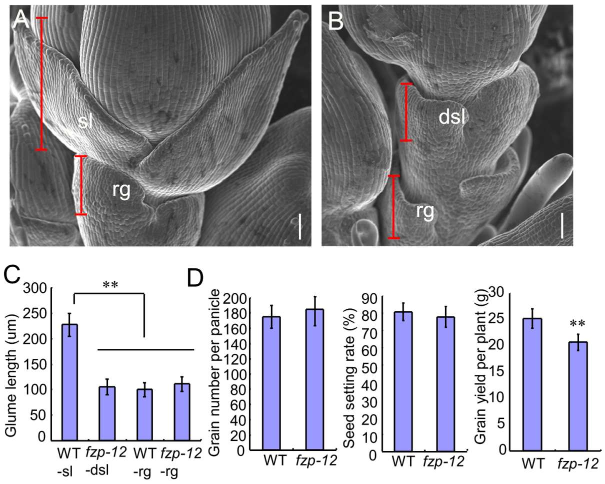


**Supplemental Figure 1.** A, wild type spikelet at Sp8 stage. B, *fzp-12* spikelet at Sp8 stage. C, length of the sterile lemma, degenerated sterile lemma, rudimentary glume in the wild type and *fzp-12* mutant. D, grain number per panicle, seed setting rate, and grain yield per plant in the wild type and *fzp-12* mutant. rg, rudimentary glume; sl, sterile lemma; dsl, degenerated sterile lemma. Red lines indicate the organ length. Bars = 50 µm. **Significant difference at $P < 0.01$ compared with the wild type by Student's *t*-test.

### Supplemental Figure 2

```
        1                                                          60
FZP     MNTRGSGSSSSSSSSQASLMAFSEPPKPASQPSPPSSPMSERPPSGRSRRRAQEPGRFLG
fzp-12  MNTRGSGSSSSSSSSQASLMAFSEPPKPASQPSPPSSPMSERPPSGRSRRRAQEPGRFLG
fzp-13  MNTRGSGSSSSSSSSQASLMAFSEPPKPASQPSPPSSPMSERPPSGRSRRRAQEPGRFLG
        61                                                        120
FZP     VRRRPWGRYAAEIRDPTTKERHWLGTFDTAQEAALAYDRAALSMKGAQARTNFVYTHAAY
fzp-12  VRRRPWGRYAAEIRDPTTKERHWLGTFDTTQEAALAYDRAALSMKGAQARTNFVYTHAAY
fzp-13  VRRRPWGRYAAEIRDSTTKERHWLGTFDTAQEAALAYDRAALSMKGAQARTNFVYTHAAY
        121                                                       180
FZP     NYPPFLAPFHAPQYAAAAAAPSSVQYGGGVGAAPHIGSYGHHHHHHHHHGHGAASGASSV
fzp-12  NYPPFLAPFHAPQYAAAAAAPSSVQYGGGVGAAPHIGSYGHHHHHHHHHGHGAASGASSV
fzp-13  NYPPFLAPFHAPQYAAAAAAPSSVQYGGGVGAAPHIGSYGHHHHHHHHHGHGAASGASSV
        181                                                       240
FZP     GECSTMPVMVPVDPHRSSMSSSLLDMDRNGHDFLFSGADDNSGYLSSVVPESCLRPRGGG
fzp-12  GECSTMPVMVPVDPHRSSMSSSLLDMDRNGHDFLFSGADDNSGYLSSVVPESCLRPRGGG
fzp-13  GECSTMPVMVPVDPHRSSMSSSLLDMDRNGHDFLFSGADDNSGYLSSVVPESCLRPRGGG
        241                                                       300
FZP     AAADHQDMRRYSDADAYGMMGLREDVDDLAQMVAGFWGGGDAADQLGACGFPASGGAADM
fzp-12  AAADHQDMRRYSDADAYGMMGLREDVDDLAQMVAGFWGGGDAADQLGACGFPASGGAADM
fzp-13  AAADHQDMRRYSDADAYGMMGLREDVDDLAQMVAGFWGGGDAADQLGACGFPASGGAADM
        301              318
FZP     VASSQGSDSYSPFSFLSH
fzp-12  VASSQGSDSYSPFSFLSH
fzp-13  VASSQGSDSYSPFSFLSH
```

**Supplemental Figure 2.** Protein sequence alignment of FZP, fzp-12, and fzp-13. Red

arrows indicate the mutated sites in the *fzp-12* and *fzp-13* mutants. *fzp-13* indicates the Cas9 knock-out plant.

## Supplemental Figure 3

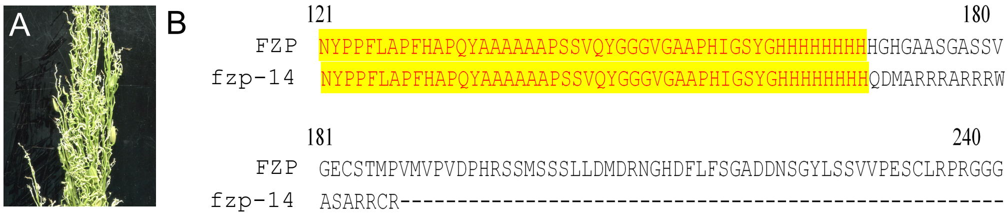


B

```
        121                                                        180
   FZP  NYPPFLAPFHAPQYAAAAAAPSSVQYGGGVGAAPHIGSYGHHHHHHHHHGHGAASGASSV
fzp-14  NYPPFLAPFHAPQYAAAAAAPSSVQYGGGVGAAPHIGSYGHHHHHHHHQDMARRRARRRW

        181                                                        240
   FZP  GECSTMPVMVPVDPHRSSMSSSLLDMDRNGHDFLFSGADDNSGYLSSVVPESCLRPRGGG
fzp-14  ASARRCR-----------------------------------------------------
```

**Supplemental Figure 3.** *fzp-14* mutant and protein sequence alignment of FZP and fzp-14. A, *fzp-14* mutant. B, protein sequence alignment of FZP and fzp-14. *fzp-14* indicates the Cas9 knock-out plant.

## Supplemental Figure 4

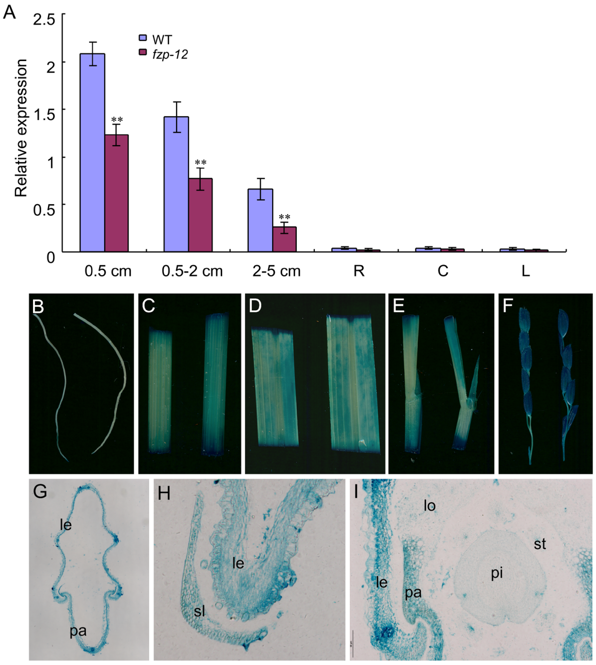


**Supplemental Figure 4.** A, *FZP* expression in different tissues as detected by qPCR. B, GUS staining of root. C, GUS staining of culm. D, GUS staining of leaf. E, GUS staining of leaf sheath. F, GUS staining of spikelet. R, root; C, culm; L, leaf. G-I, Cross-section of the stained spikelets. G, Cross-section of hull. H, Cross-section of sterile lemma. I, Cross-section of inner floral organs. 0.5, young panicles (≤0.5 cm);

0.5-2, young panicles (0.5-2 cm); 2-5, young panicles (2-5 cm). le, lemma; pa, palea; sl, sterile lemma; st, stamen; pi, pistil. Error bars indicate SD. **Significant difference at $P < 0.01$ compared with the wild type by Student's *t*-test.

**Supplemental Figure 5**

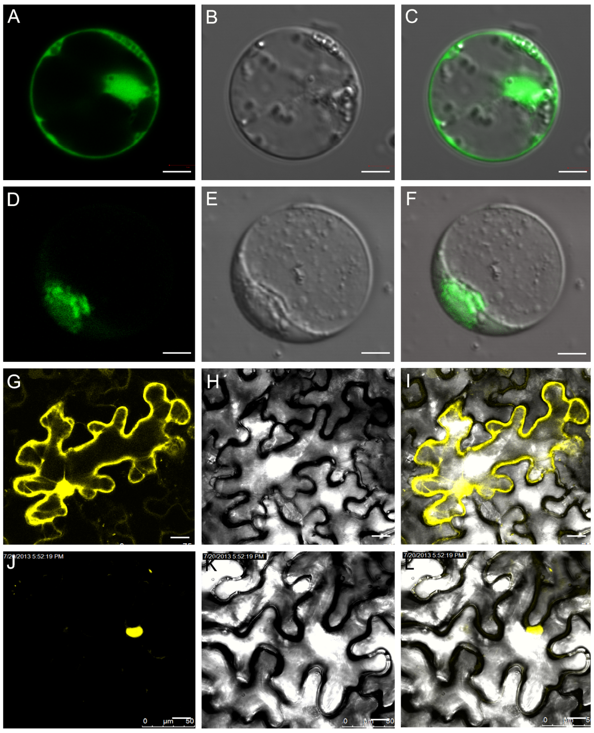


**Supplemental Figure 5.** Subcellular localization of the FZP protein. A-C, single GFP protein in rice protoplasts. D-F, FZP-GFP in rice protoplasts. G-I, single YFP protein in tobacco (*Nicotiana tabacum*) epidermal cells. J-L, FZP-YFP in tobacco (*Nicotiana tabacum*) epidermal cells. Bars = 5 um in A-F; 10um in G-L.

**Supplemental Figure 6**

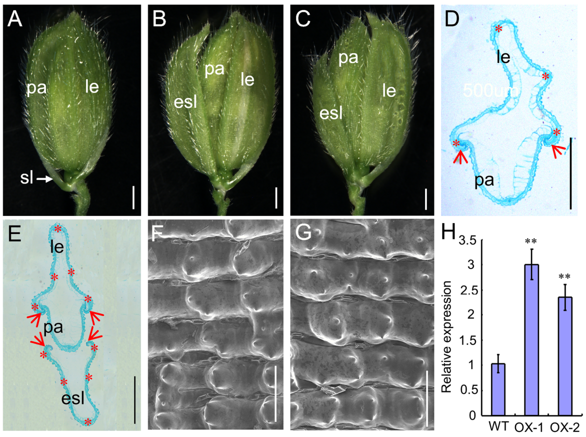


**Supplemental Figure 6.** A, wild type spikelet. B, spikelet in the *FZP* over-expressing line 1. C, spikelet in the *FZP* over-expressing line 2. D, histological analysis of wild type spikelet. E, histological analysis of spikelet in the *FZP* over-expressing plant. F, epidermal surface of the lemma in the wild type. G, epidermal surface of the sterile lemma in the *FZP* over-expressing plant. H, *FZP* expression analysis in the wild type and *FZP* over-expressing plants. esl, enlarged sterile lemma; sl, sterile lemma; le, lemma; pa, palea. Red stars indicate the vascular bundle. Red arrows indicate the inward hook-like structure. Error bars indicate SD. **Significant difference at $P <$ 0.01 compared with the wild type by Student's *t*-test. Bars = 1000 µm in A-C; 500um in D and E; 100um in F and G.

**Supplemental Figure 7**

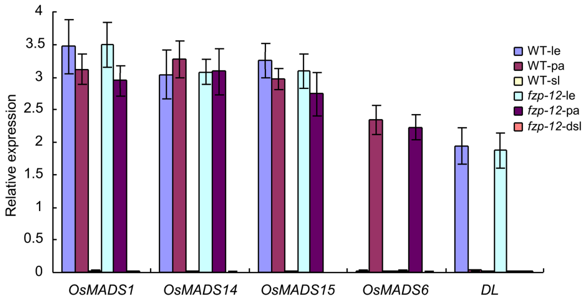


**Supplemental Figure 7.** Expression of floral organ identity genes in the wild type and *fzp-12* mutant. le, lemma; pa, palea; sl, sterile lemma; dsl, degenerated sterile lemma.

**Supplemental Figure 8**

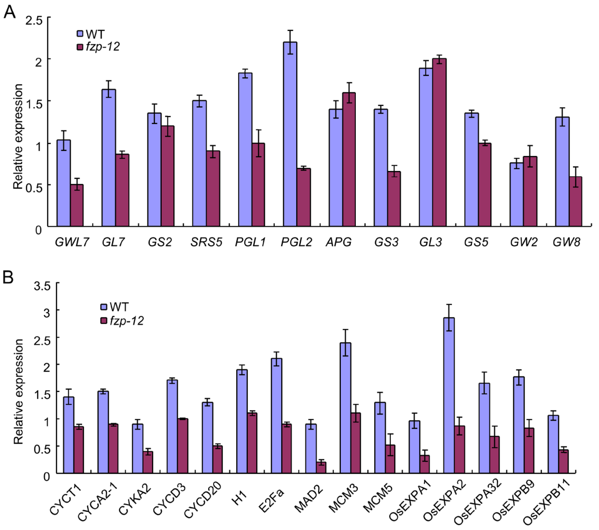


**Supplemental Figure 8.** Expression levels of related genes in wild-type and *fzp-12* mutant. A, Expression analysis of related genes influencing grain size in young panicles. B, Expression analysis of cell proliferation and expansion-related genes in young panicles. Error bars indicate SD. **Significant difference at $P < 0.01$ compared with the wild type by Student's *t*-test.

**Supplemental Figure 9.** Protein sequence alignment of AP2/ERF genes.

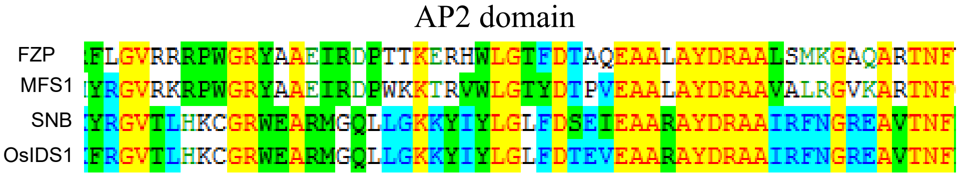


## Supplemental Tables

## Supplemental Table 1

| Cross combination | F1 | | F2 | | $X^2$ (3:1) | P-value |
|---|---|---|---|---|---|---|
| | Wild type | *fzp-12* | Wild type | *fzp-12* | | |
| *fzp-12*/NJ6 | 57 | 0 | 3612 | 1189 | 0.1406 | 0.7707 |
| NJ6/*fzp-12* | 48 | 0 | 3025 | 1043 | 0.8863 | 0.3465 |

**Supplemental Table 1.** Genetic analysis of the mutant phenotypes of *fzp-12*.

**Supplemental Table 2.** Primers used in the study.

| Purpose | Primer name | Sequence |
|---|---|---|

| | | |
|---|---|---|
| Mapping | B7-13-1F | ACAGTATCCAAGGCCCTGG |
| | B7-13-1R | CACGTGAGACAAAGACGGAG |
| | A7-29-1F | AGTTTTCCTTTCTTTTCTAC |
| | A7-29-1R | CCTCAGCATGATACCCAC |
| | A7-33-1F | GCCCAAGATTATGAGA |
| | A7-33-1R | AAGCTCCGACAATACA |
| | A7-3-1F | GAGTTCAAGGAAGCACAAAA |
| | A7-3-1R | CAATGAGACTGTTGCTAACC |
| | A7-9-1F | TGAGGAGGAGGAGATGGTCG |
| | A7-9-1R | AGGTGCCCTTTGCAGTTTGA |
| | A7-13-1F | TGCTACAAGTACGTGCTTTT |
| | A7-13-1R | GTGGTTGGAGGCGATTTTAT |
| | A7-25-1F | TGCGTGGTGCAGCATTTGTA |
| | A7-25-1R | GGCATCACATTGAGCAAGAAA |
| | A7-26-1F | ACGTGCTTTTCTTGTACCTT |
| | A7-26-1R | TTTGTGGTTGGAGGCGATTT |
| | S2-1F | CCTTAAGCTTGCATGGAG |
| | S2-1R | GGTGGCCGGTGAAAAGT |
| | S14-1F | GAGCTTTGCCTACAAGG |
| | S14-1R | CGGGGAACAATGTAACAC |
| qRT-PCR | *FZP*-1F | GCATGGCTAATCACGCACTTT |
| | *FZP*-1R | CCAAGCCACTCTTCTTGTTCG |
| | *OsMADS1*-1F | |
| | *OsMADS1*-1R | TGATGGTGAGCATGAGGGTG |
| | *OsMADS14*-1F | CCATTAACGAGCTTCAACGG |
| | *OsMADS14*-1R | TGGTATGGATCTGAAGCCTCC |
| | *OsMADS15*-1F | AGTACGCCACTGACTCCAGG |
| | *OsMADS15*-1R | TGCTGGCCCCTCACATTC |
| | *OsMADS6*-1F | CCAACAATGCACTTTCTGAAAC |
| | *OsMADS6*-1R | GGAGGCTTGCTGCATGGC |
| | *DL*-1F | CCCATCTGCTTACAACCGCTT |
| | *DL*-1R | GTTGGAGGTGGAAACCGTCG |
| | *G1*-1F | GGCGTCTACTTGCCATTTCTG |
| | *G1*-1R | TCGATCAGCATCAAAGCACAG |
| | *ACTIN*-1F | AGCAACTGGGATGATATGGA |
| | *ACTIN*-1R | CAGGGCGATGTAGGAAAGC |
| GUS staining | pro*FZP*-GUS-1F | CAAGAGGAATTAACAGTGCTAATTTC |
| | pro*FZP*GUS-1R | GTGTTCATGACCAATGCAATATTG |
| In Situ Hybridization | *G1*H-1F | CGCCTACGACGAGCGTCA |
| | *G1*SP6-1R | AGATTTAGGTGACACTATAGAAAGCAAC CACAGTGAGAAGCACAG |
| | *DL*H-1F | GGGCCGCAATGGATCTCGTG |
| | *DL* SP6-1R | AGATTTAGGTGACACTATAGAATCACAA |

| | | |
|---|---|---|
| | | CGAAGGGTGCTCT |
| Complementation test | *FZP*com-1F | CAAGAGGAATTAACAGTGCTAATTTC |
| | *FZP*com-1R | CCACCACCTGCGTGTTATATG |
| | *FZP*OE-1 F | ATGAACACTCGAGGCAGCGG |
| | *FZP*OE-1 R | TCAATGGGAGAGGAAGCTGAATG |
| Over-expressing | *FZP*OE-1F | ATGAACACTCGAGGCAGCGG |
| | *FZP*OE-1R | TCAATGGGAGAGGAAGCTGAATG |
| EMSA probe (specific) | GCC-1F | CATAAGAGCCGCCACTCATAAGAGCCGCC ACTCATAAGAGCCGCCACT |
| | GCC-1R | GTATTCTCGGCGGTGAGTATTCTCGGCGG TGAGTATTCTCGGCGGTGA |
| EMSA probe (non-specific) | GCC-2F | CATAAGATACTTGACTCATAAGATACTTGA CTCATAAGATACTTGACT |
| | GCC-2R | GTATTCTATGAACTGAGTATTCTATGAACT GAGTATTCTATGAACTGA |
| EMSA probe (specific) | DRE-1F | ATACTACCGACATGAGATACTACCGACAT GAG ATACTACCGACATGAG |
| | DRE-1R | TATGATGGCTGTACTCTATGATGGCTGTAC TCTATGATGGCTGTACTC |
| EMSA probe (non-specific) | DRE-2F | ATACGTCTTTGACGAGATACGTCTTTGAC GAGATACGTCTTTGACGAG |
| | DRE-2R | TATGCAGAAACTGCTCTATGCAGAAACTG CTC TATGCAGAAACTGCTC |
